# Author Correction: Causal networks of phytoplankton diversity and biomass are modulated by environmental context

**DOI:** 10.1038/s41467-022-33702-1

**Published:** 2022-10-05

**Authors:** Chun-Wei Chang, Takeshi Miki, Hao Ye, Sami Souissi, Rita Adrian, Orlane Anneville, Helen Agasild, Syuhei Ban, Yaron Be’eri-Shlevin, Yin-Ru Chiang, Heidrun Feuchtmayr, Gideon Gal, Satoshi Ichise, Maiko Kagami, Michio Kumagai, Xin Liu, Shin-Ichiro S. Matsuzaki, Marina M. Manca, Peeter Nõges, Roberta Piscia, Michela Rogora, Fuh-Kwo Shiah, Stephen J. Thackeray, Claire E. Widdicombe, Jiunn-Tzong Wu, Tamar Zohary, Chih-hao Hsieh

**Affiliations:** 1 National Center for Theoretical Sciences, Taipei, 10617 Taiwan; 2 Research Center for Environmental Changes, Academia Sinica, Taipei, 11529 Taiwan; 3 Faculty of Advanced Science and Technology, Ryukoku University, Otsu, Shiga, 520-2194 Japan; 4 Institute of Oceanography, National Taiwan University, Taipei, 10617 Taiwan; 5 Center for Biodiversity Science, Ryukoku University, Otsu, Shiga, 520-2194 Japan; 6 Health Science Center Libraries, University of Florida, Gainesville, FL 32611 USA; 7 Univ. Lille, CNRS, Univ, Littoral Côte D’Opale, IRD, UMR 8187, LOG— Laboratoire D’Océanologie et de Géosciences, Station Marine de Wimereux, F- 59000 Lille, France; 8 Leibniz Institute of Freshwater Ecology and Inland Fisheries, IGB, 12587 Berlin, Germany; 9 Freie Universität Berlin, Department of Biology, Chemistry and Pharmacy, 14195 Berlin, Germany; 10 National Research Institute for Agriculture, Food and Environment (INRAE), CARRTEL, Université Savoie Mont Blanc, 74200 Thonon les Bains, France; 11 Centre for Limnology, Institute of Agricultural and Environmental Sciences, Estonian University of Life Sciences, Kreutzwaldi 5D, 51014 Tartu, Estonia; 12 Department of Ecosystem Studies, School of Environmental Science, The University of Shiga Prefecture, Hikone, 522-8533 Shiga Japan; 13 Kinneret Limnological Laboratory, Israel Oceanographic & Limnological Research, P.O. Box 447, 14950 Migdal, Israel; 14 Biodiversity Research Center, Academia Sinica, Taipei, 11529 Taiwan; 15 UK Centre for Ecology & Hydrology, Lancaster Environment Centre, Library Avenue, Bailrigg, Lancaster, Lancashire LA1 4AP UK; 16 Lake Biwa Environmental Research Institute, Otsu, 520-0022 Japan; 17 Faculty of Environment and Information Sciences, Yokohama National University, Yokohama, 240-8502 Kanagawa Japan; 18 Department of Environmental Science, Faculty of Science, Toho University, Funabashi, Chiba, 274-8510 Japan; 19 Research Center for Lake Biwa & Environmental Innovation, Ritsumeikan University, Kusatsu, 525-0058 Shiga Japan; 20 Biodiversity Division, National Institute for Environmental Studies, 16-2 Onogawa, Tsukuba, Ibaraki 305-8506 Japan; 21 CNR Water Research Institute (IRSA), L.go Tonolli 50, 28922 Verbania, Pallanza Italy; 22 Plymouth Marine Laboratory, Prospect Place, West Hoe, Plymouth, PL1 3DH UK; 23 Institute of Ecology and Evolutionary Biology, Department of Life Science, National Taiwan University, Taipei, 10617 Taiwan

**Keywords:** Biodiversity, Ecology, Ecological networks, Ecosystem ecology

**Correction to**: *Nature Communications* 10.1038/s41467-022-28761-3, published online 03 March 2022

The original version of this Article contained an error in the Acknowledgements, which incorrectly read: “Data for Lake Võrtsjärv were provided by Estonian Environment Agency and by the Centre for Limnology at Estonian University of Life Sciences funded by the Estonian Research Council grants PRG 1167 and PRG709, and by the European Union’s Horizon 2020 research and innovation programme under grant agreement No. 95196.” The correct version replaces this sentence with “Data for Lake Võrtsjärv were provided by Estonian Environment Agency and by the Centre for Limnology at Estonian University of Life Sciences funded by the Estonian Research Council grants PRG 1167 and PRG709. This project has received funding from the European Union’s Horizon 2020 research and innovation programme under grant agreement No 951963.” This has now been corrected in both the HTML and the PDF versions of the Article.

