# Supplementary material for: Causal networks of phytoplankton diversity and biomass are modulated by environmental context
Source: Nat Commun. 2022 Mar 3;13:1140. doi: 10.1038/s41467-022-28761-3 (PMC8894464; doi:10.1038/s41467-022-28761-3)
Supplement: Supplementary file 1 — Supplementary Information [file 41467_2022_28761_MOESM1_ESM.pdf]

Supplementary Materials for

Causal networks of phytoplankton diversity and biomass are modulated by environmental context

Chun-Wei Chang, Takeshi Miki, Hao Ye, Sami Souissi, Rita Adrian, Orlane Anneville, Helen Agasild, Syuhei Ban, Yaron Be'eri-Shlevin, Yin-Ru Chiang, Heidrun Feuchtmayr, Gideon Gal, Satoshi Ichise, Maiko Kagami, Michio Kumagai, Xin Liu, Shin-Ichiro S. Matsuzaki, Marina M. Manca, Peeter Nõges, Roberta Piscia, Michela Rogora, Fuh-Kwo Shiah, Stephen J. Thackeray, Claire E. Widdicombe, Jiunn-Tzong Wu, Tamar Zohary, Chih-hao Hsieh*

Correspondence to: chsieh@ntu.edu.tw

This PDF file includes:

Supplementary Figs. S1 to S14

Supplementary Tables S1 to S3

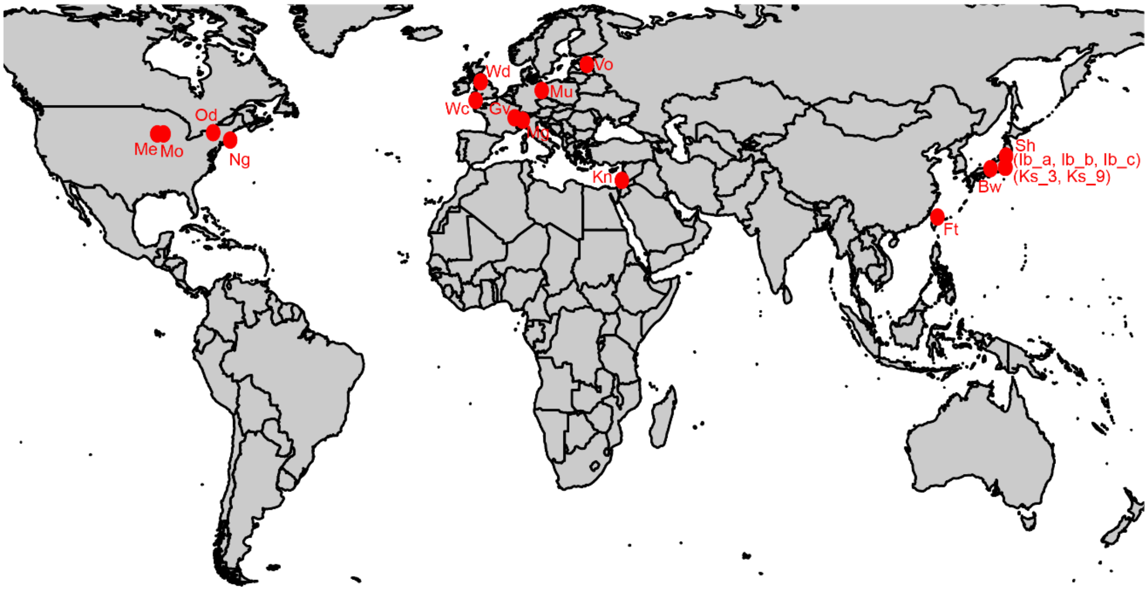

Figure S1 | Locations of long-term ecological monitoring systems labeled by red points, including 16 aquatic ecosystems with a total of 19 sites, including: 1) Lake Biwa, Japan (Bw); 2) Feitsui Reservoir, Taiwan (Ft); 3) Lake Geneva, France/Swiss (Gv); 4) Lake Inba, Japan involving three independent stations (Ib_a, Ib_b, and Ib_c); 5) Lake Kasumigura, Japan involving two independent stations (Ks_3 and Ks_9); 6) Lake Kinneret, Israel (Kn); 7) Lake Maggiore, Italy (Mg); 8) Lake Mendota, USA (Me); 9) Lake Monona, USA (Mo); 10) Müggelsee, Germany (Mu); 11) Narragansett Bay, USA (Ng); 12) Lake Oneida, USA (Od); 13) Shin River, Japan (Sh); 14) Lake Vortsjarv, Estonia (Vo); 15) Station L4, Western English Channel, England (Wc); 16) Windermere, England (Wd).

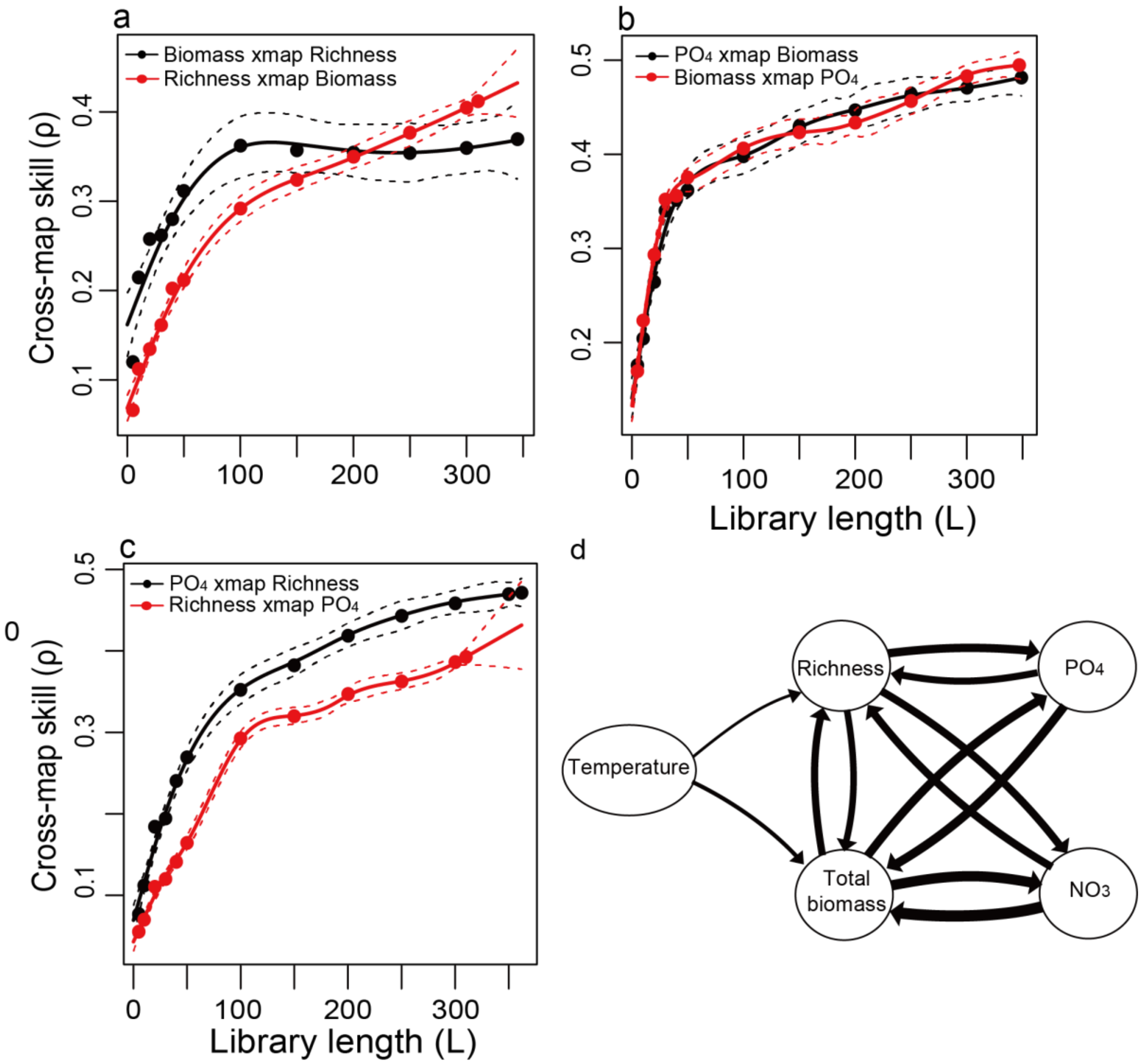

Figure S2 | Example of causality testing among phytoplankton diversity, biomass, and environmental factors in a natural aquatic ecosystem. (Panels (**a**)-(**c**)) Examples of convergent cross mapping of phytoplankton species diversity (richness), phytoplankton biomass (chlorophyll *a* concentration), and phosphate concentration in Lake Kasumigaura Station 9. The solid line represents the fitted regression spline, with the dashed lines enclosing the 95% confidence interval. The skill of CCM estimates (ρ) increasing with time series length indicates a causal relationship. Panel (**d**) summarizes the causal network reconstructed by pairwise CCM analyses. The thickness of each arrow indicates the linkage strength, i.e., the cross-map skill obtained under the maximal library length, $\rho(L_{max})$. Analogously, the causal network consisted of 12 causal links was examined for each of the 19 sites.

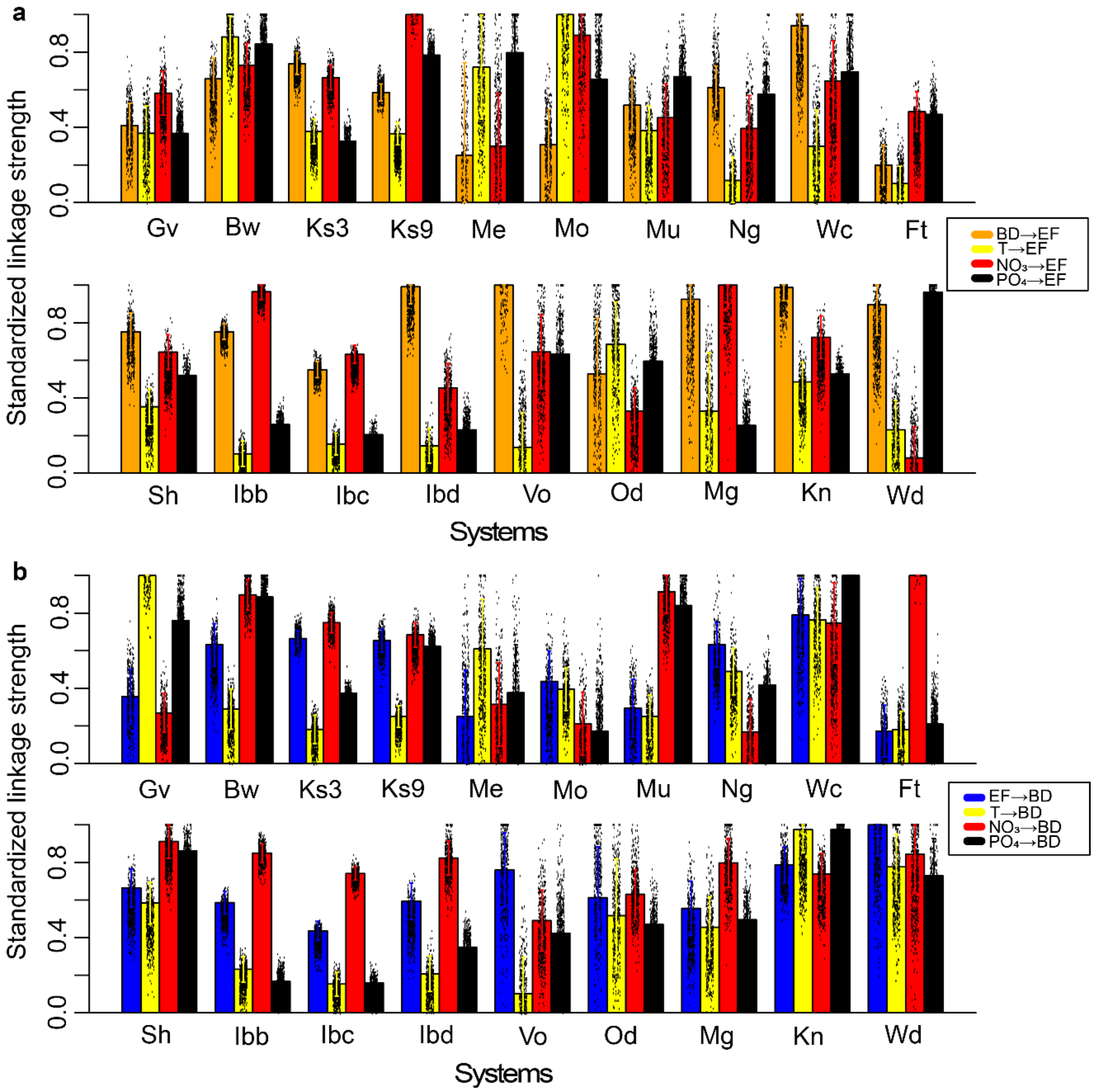

Figure S3 | Standardized linkage strength of determinants for phytoplankton biomass (EF) (a) and species richness (BD) (b) for each system. Bar height presents the standardized linkage strength estimated by CCM with the error bar presenting its standard error (± 1 SEM). Here, the standard error was estimated from the sampling distribution of the strengths generated from 500 times data resampling (displaying as dots; n=500).

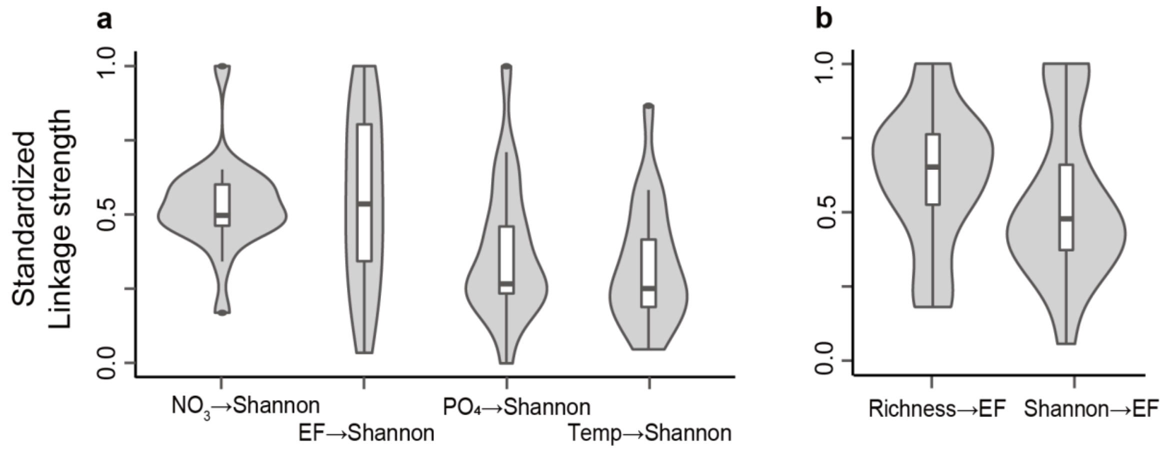

Figure S4 | Relative strengths of various causal drivers associated with phytoplankton Shannon diversity. Panel (**a**) is comparable with Fig. 2b, revealing standardized linkage strengths of various causal variables affecting phytoplankton diversity based on Shannon diversity. All statistics were calculated from the 19 independent sites (n=19) and depicted as joint violins and box-plots to present the empirical distribution that labels the maxima and minima at the top and bottom of the violins, respectively, and shows 25, 50 and 75% quantiles in the boxes with whiskers presenting at most 1.5*interquartile range. Our analysis revealed that NO_3 remained an important driver on Shannon diversity, whereas effects of phytoplankton biomass (EF) became more important. In that regard, changes in total phytoplankton biomass might be better associated with dominance of some phytoplankton species, which in turn influences the diversity index considering species relative composition. Nonetheless, (**b**) the effects of Shannon diversity on phytoplankton biomass were relatively weaker than species richness effects and thus were not included in the main results.

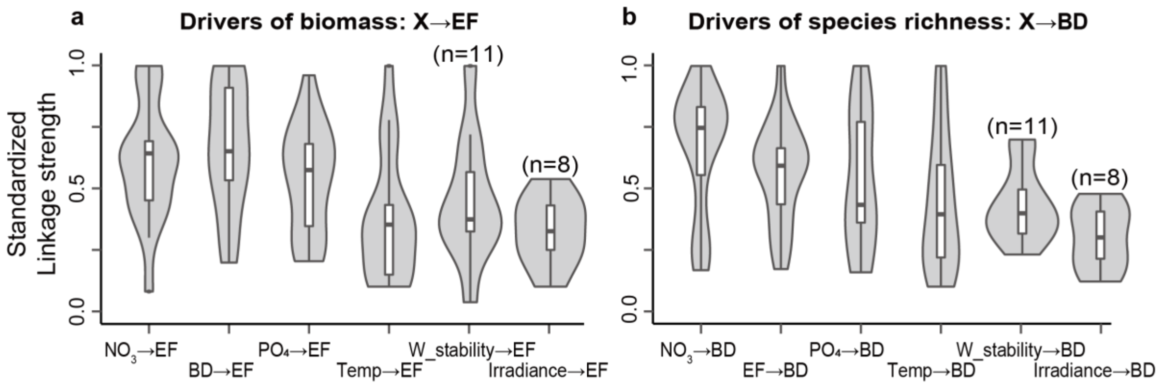

Figure S5 | Relative strengths of various drivers, including irradiance and water column stability. Panels (**a**) and (**b**) are analogies of Fig. 2a and 2b, revealing standardized linkage strengths of causal variables affecting (**a**) phytoplankton biomass (EF) and (**b**) species richness (BD). Here, we additionally considered the effects of two exogenous environmental drivers, water column stability (denoted as "W_stability") and irradiance. All statistics were calculated from the 19 independent sites (n=19), except for causal strengths of water stability (n=11) and irradiance (n=8) due to data availability (Table S3). We summarized these statistics as joint violins and box-plots to present the empirical distribution that labels the maxima and minima at the top and bottom of the violins, respectively, and shows 25, 50 and 75% quantiles in the boxes with whiskers presenting at most 1.5*interquartile range. Our analyses revealed that the causal strength of water column stability and irradiance was on average weaker than that of diversity and nutrients. However, our analyses for water column stability and irradiance remained inconclusive, based on the current datasets because water column stability and irradiance datasets were not available for every system. This made it difficult to compare these effects to those of other factors, for which we had complete datasets from 19 systems.

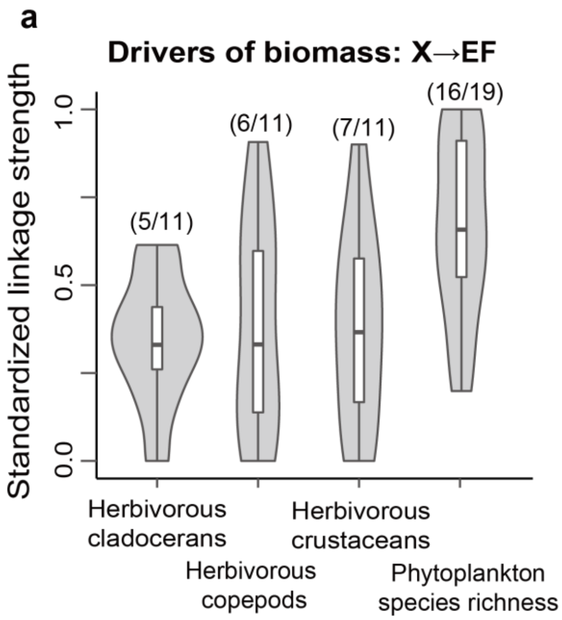

Figure S6 | Causal strengths of zooplankton drivers on ecosystem functioning and species diversity in phytoplankton communities. Panels (**a**) and (**b**) are comparable with Fig. 2a and 2b, revealing standardized linkage strengths of causal variables affecting (**a**) phytoplankton biomass (EF) and (**b**) species richness (BD). We examined the consumer effects of three zooplankton categories: i) herbivorous cladocerans, ii) herbivorous copepods, and iii) herbivorous crustaceans (See details in Methods). Statistics were calculated from 11 independent sites (n=11) for zooplankton analysis due to data availability (Table S3) but from all 19 sites (n=19) for examining species richness effects on biomass (**a**) and nitrate effects on species richness (**b**). We summarized these statistics as joint violins and box-plots to present the empirical distribution that labels the maxima and minima at the top and bottom of the violins, respectively, and shows 25, 50 and 75% quantiles in the boxes with whiskers presenting at most 1.5*interquartile range. The two numbers within the parentheses (*S*/*N*) above each violin plot report the number of significant results in CCM (*S*) and the number of analyzed systems (*N*). On average, the causal effects of zooplankton on EF and BD were significant in a majority of analyzed systems but not as strong as the effects of phytoplankton species richness and nitrates, respectively. It is noteworthy that the causal effects of herbivorous cladocerans were, though not as strong as nutrient effects, still an important driver determining phytoplankton species richness, in which causal effects were significant in 10 of the 11 analyzed systems.

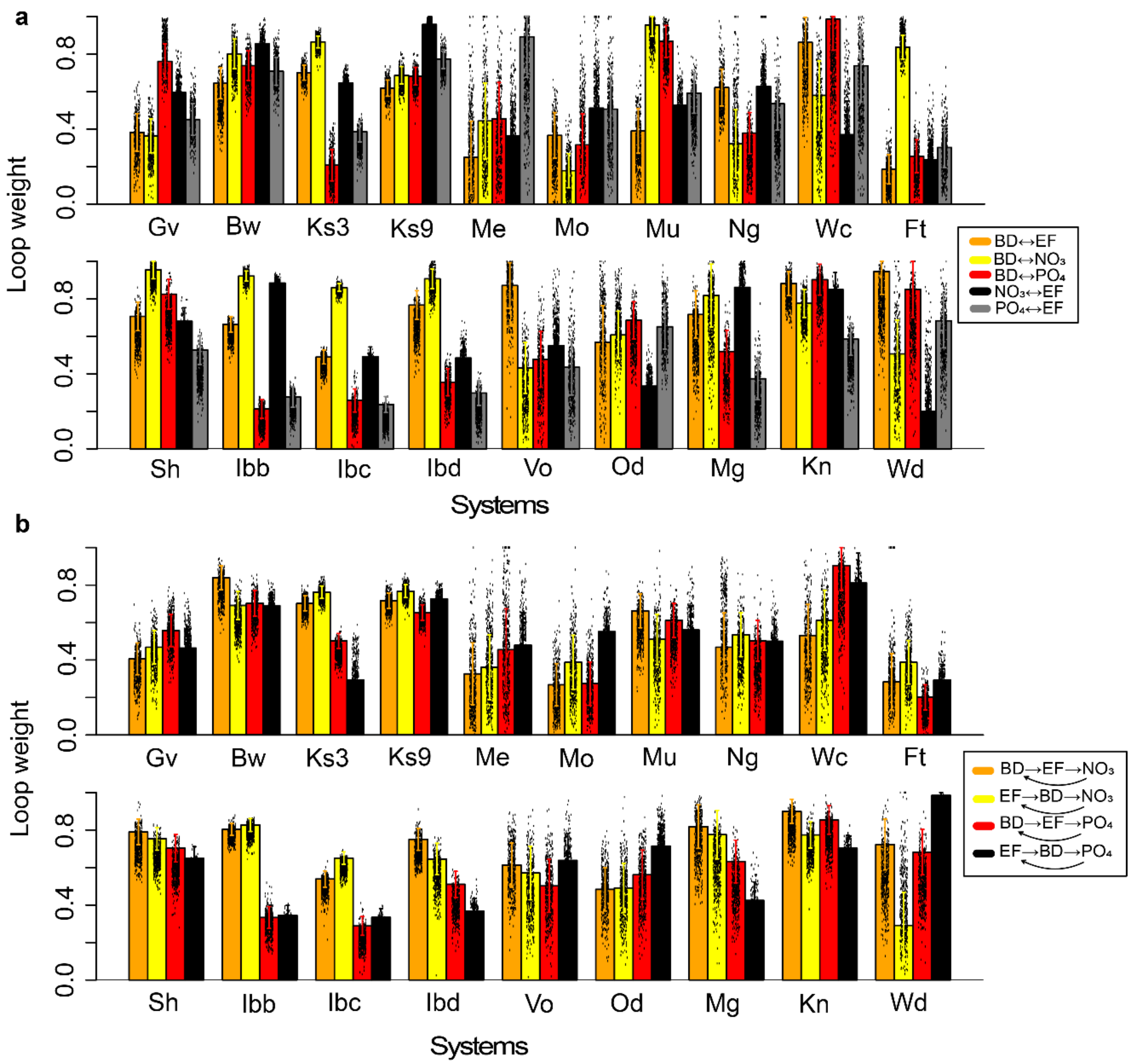

Figure S7 | Loop weights of (a) pairwise feedbacks and (b) triangular feedbacks for each system. BD indicates phytoplankton species richness; EF indicates phytoplankton biomass. Bar height presents the loop weight estimated by CCM with the error bar presenting its standard error (± 1 SEM). Here, the standard error was estimated from the sampling distribution of the loop weights generated from 500 times data resampling (displaying as dots; n=500).

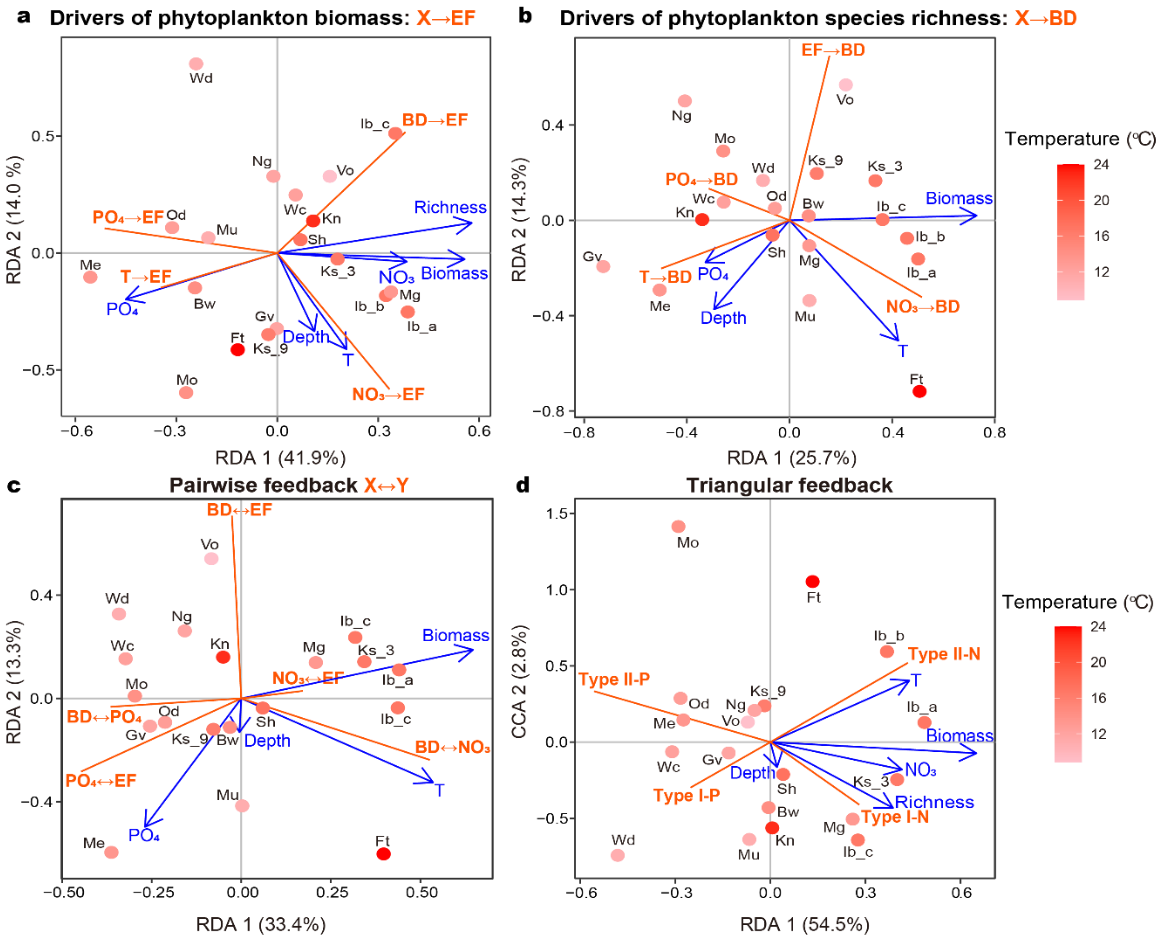

Figure S8 | Multivariate ordination illustrating associations between the long-term averages of environmental factors (blue) and quantitative network modules with colour scales (red) based on long-term water temperature. This is an analogue of Fig. 4 that included the strength of links affecting (**a**) phytoplankton biomass (X→EF; red) and (**b**) species richness (X→BD), and the loop weight of (**c**) pairwise feedbacks and (**d**) triangular feedbacks.

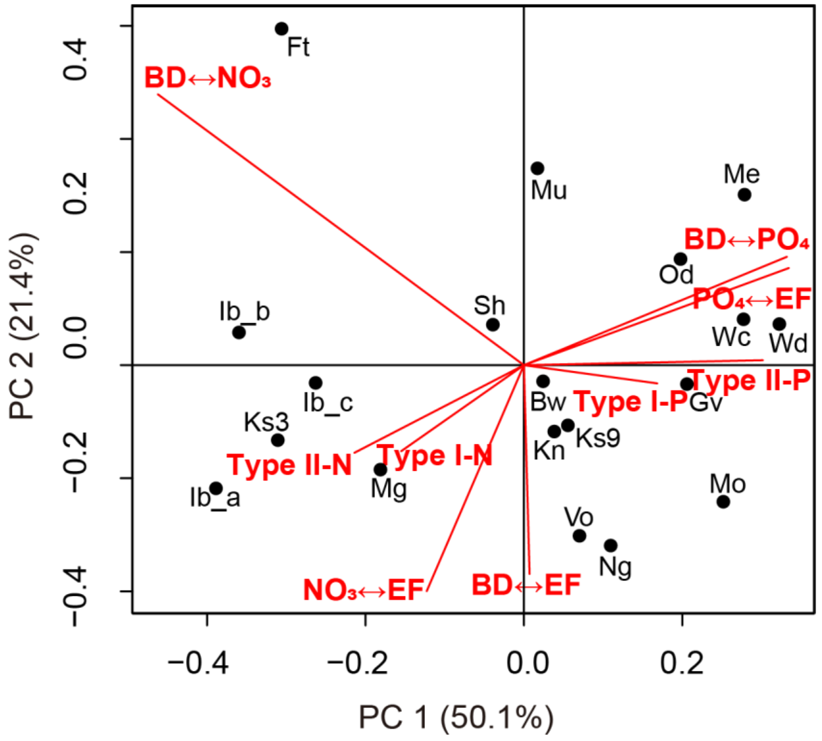

Figure S9 | Triangular feedbacks were statistically distinguishable from pairwise feedbacks. Principle component analysis (PCA) based on standardized loop weights of pairwise feedbacks and triangular feedbacks indicated that the cross-system variations in the strength of triangular feedbacks (Type I-N, Type II-N, Type I-P, Type II-P) were different from the variations of pairwise feedbacks (e.g., BD↔EF).

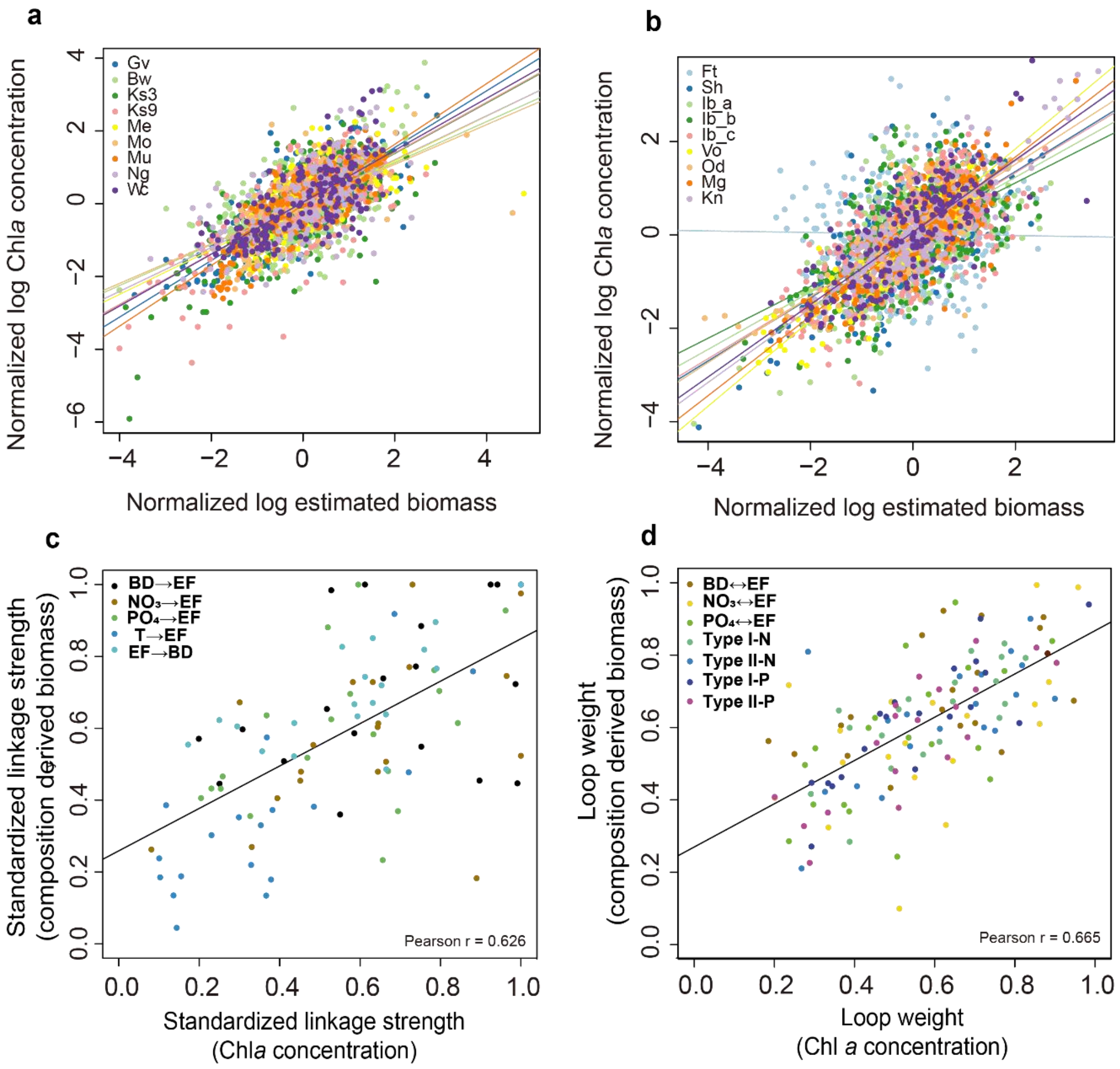

Figure S10 | Quantification of causal strength was robust to the use of the alternative biomass measure converted from phytoplankton composition data. In many of our studied systems, the biomass measures were obtained from composition data using species-specific conversion factors. These conversion factors were assumed fixed in absence of detailed measurement in individual cell size and estimated by various geometrical models that differed among studied systems. (**a-b**) Composition converted biomass (log transformed) were highly correlated with chlorophyll *a* concentration (Chl*a*) in most systems (mean Pearson r = .668). (**c**) Causal strengths and (**d**) loop weights derived from analysing composition converted biomass (log transformed) were highly correlated with the strengths derived from analysing Chl*a* (Pearson r=.626 and .665, respectively).

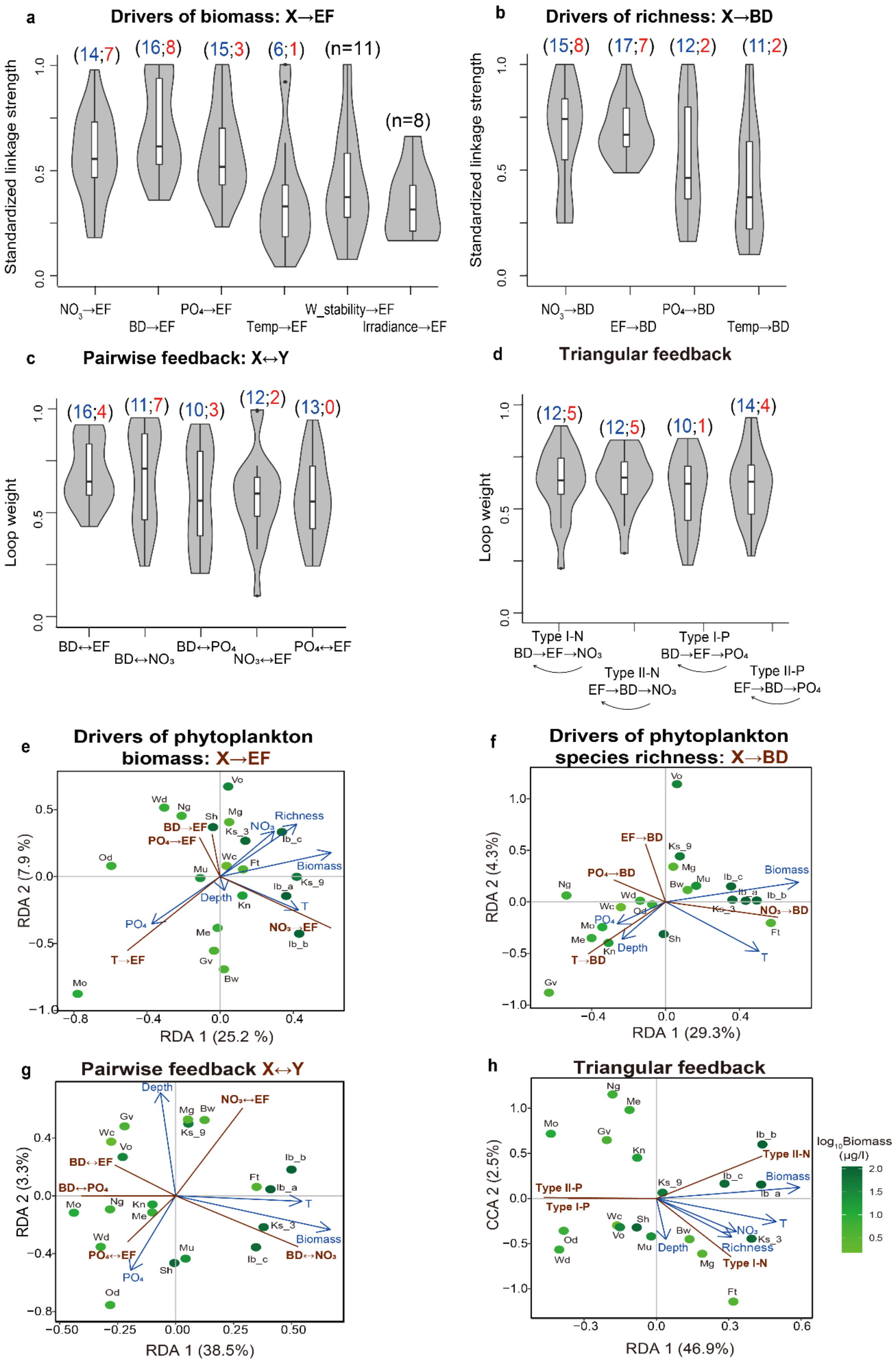
a Drivers of biomass: X→EF
Standardized linkage strength
(14;7) (16;8) (15;3) (6;1) (n=11) (n=8)
NO₃→EF BD→EF PO₄→EF Temp→EF W_stability→EF Irradiance→EF
b Drivers of richness: X→BD
(15;8) (17;7) (12;2) (11;2)
NO₃→BD EF→BD PO₄→BD Temp→BD
c Pairwise feedback: X↔Y
Loop weight
(16;4) (11;7) (10;3) (12;2) (13;0)
BD↔EF BD↔NO₃ BD↔PO₄ NO₃↔EF PO₄↔EF
d Triangular feedback
(12;5) (12;5) (10;1) (14;4)
Type I-N BD→EF→NO₃
Type II-N EF→BD→NO₃
Type I-P BD→EF→PO₄
Type II-P EF→BD→PO₄
e Drivers of phytoplankton biomass: X→EF
RDA 1 (25.2 %)
RDA 2 (7.9 %)
f Drivers of phytoplankton species richness: X→BD
RDA 1 (29.3%)
RDA 2 (4.3%)
g Pairwise feedback X↔Y
RDA 1 (38.5%)
RDA 2 (3.3%)
h Triangular feedback
RDA 1 (46.9%)
CCA 2 (2.5%)
log10Biomass (μg/l)

Figure S11 | The findings of main analyses were robust to the use of the alternative biomass measure converted from phytoplankton composition data. The findings based on biomass measures were obtained from composition data using species-specific conversion factors regarding (**a-d**), and the relative importance among various causal links and feedback (an analogue of Fig. 2) and (**e-h**) the statistical associations with environmental factors (an analogue of Fig. 4) were qualitatively similar with our main findings based on analysing Chl*a*. All statistics were calculated from the 19 independent sites (n=19), except for causal strengths of water stability (n=11) and irradiance (n=8) on biomass (**a**) due to data availability (Table S3). We summarized these statistics as joint violins and box-plots to present the empirical distribution that labels the maxima and minima at the top and bottom of the violins, respectively, and shows 25, 50 and 75% quantiles in the boxes with whiskers presenting at most 1.5*interquartile range. Specifically, species richness was still an important driver affecting composition converted biomass compared to the other environmental factors. In addition, the RDA ordinations (**e-h**) based on the same sets of environmental variables manifested similar patterns presented in Fig. 4, but explained less variations compared to ordinations based on Chl*a* [proportions of explained variations (RDA 1+RDA 2) = 33.1, 33.6, 41.8, and 49.4% were less than those presented in Fig. 4 =55.9, 40.0, 46.7, and 57.3%, respectively].

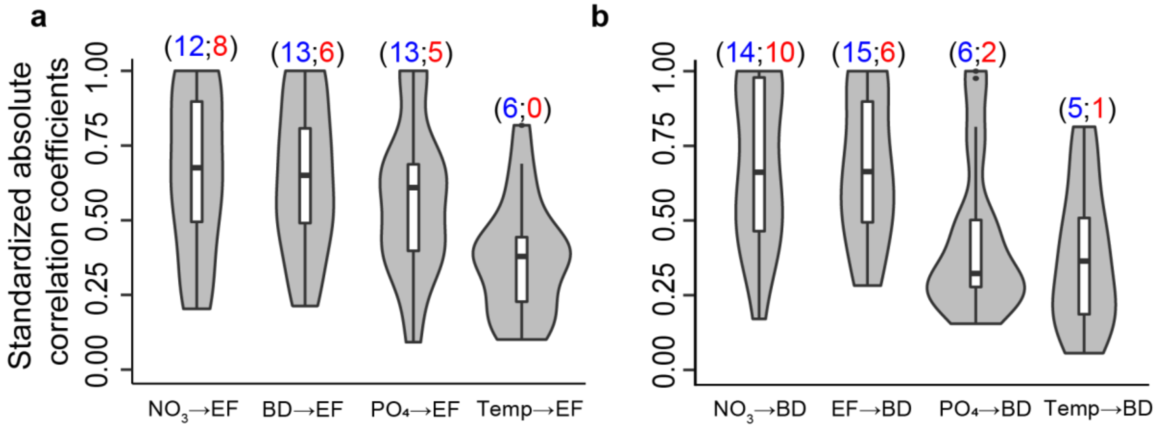

Figure S12 | Relative importance of various determinants for phytoplankton biomass (a) and species richness (b) evaluated by the absolute value of correlation coefficient. Here, the absolute values of correlation coefficients were used as a proxy of interaction strength. Correlation coefficients were estimated from the cross-correlation analysis using the same data as CCM analyses (detrended and deseasoned). In this cross-correlation analysis, we considered at most 3-month lag effects for each determinant. Similar to Fig. 2, we standardized these correlation coefficients by dividing the maximal value within each system to eliminate systematic differences among study sites. All the derived statistics were calculated from the 19 independent sites (n=19) and depicted as joint violins and box-plots to present the empirical distribution that labels the maxima and minima at the top and bottom of the violins, respectively, and shows 25, 50 and 75% quantiles in the boxes with whiskers presenting at most 1.5*interquartile range. The two numbers within the parentheses (S; R_1) above each violin plot represent the number of significant results (S; in blue) and number of systems in which a particular module had the greatest strength (i.e., rank 1; R_1; in red).

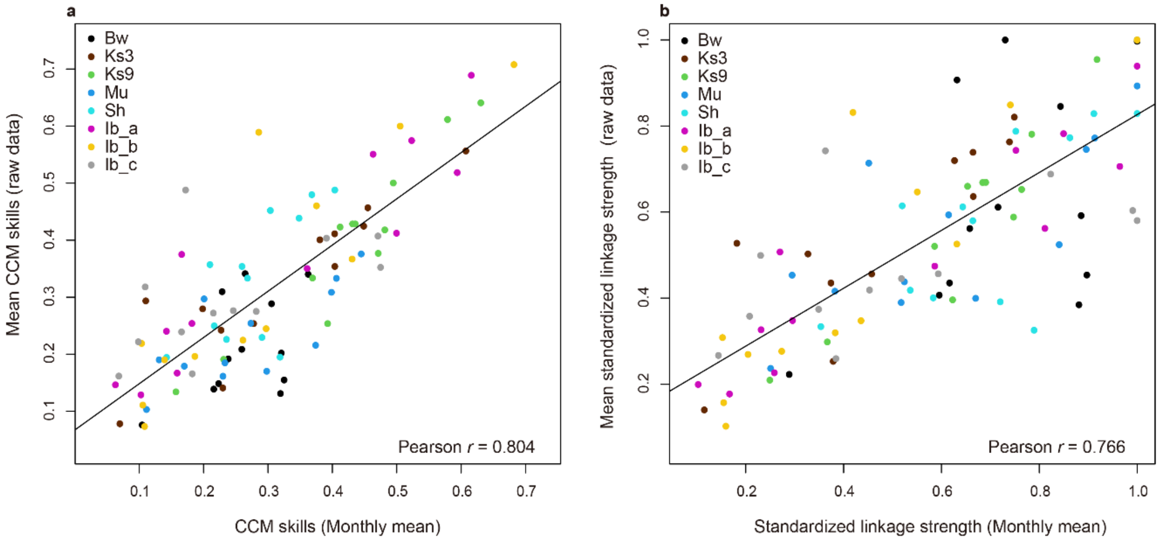

Figure S13 | Estimated causal strengths were robust to data integration that averaged over the observations on monthly scale. To investigate the influences of data integration on CCM estimates, we compiled time series data using non-integrated measurements while fixed the time scale (i.e., monthly) of analysis. To do so, we designed two ways to generate monthly time series data from the datasets with at least biweekly resolution. In each of the two time series, non-integrated data was collected from either the early half (day≦15) or later half (day>15) in a month, respectively.

Ideally, this way of data compilation yields two independent monthly time series composed of non-integrated measurements if the field sampling was arranged very regularly with at least biweekly frequency. However, considering the missing data issue, we were able to carry out such analysis from only eight systems, in which at least 80% of data points were available, including Lake Biwa (Bw), two stations in Lake Kasumigaura (Ks_3& Ks_9), Shin River (Sh), three stations in Lake Inba (Ib_a, Ib_b, & Ib_c), and Müggelsee (Mu). However, only one monthly time series can be compiled in Bw, Ks_3, and Ks_9. Based on the analysis of non-integrated datasets, causal strengths measured by averaged (a) CCM skills and (b) standardized linkage strength were still strongly correlated with the strengths estimated by monthly integrated data. This finding indicates that the results based on monthly integrated data are similar with the averaged results computed from analysing the two time series composed of non-integrated data points. Overall, our main findings were robust to the data compilation (i.e., averaging), provided that the time scale of analysis is fixed.

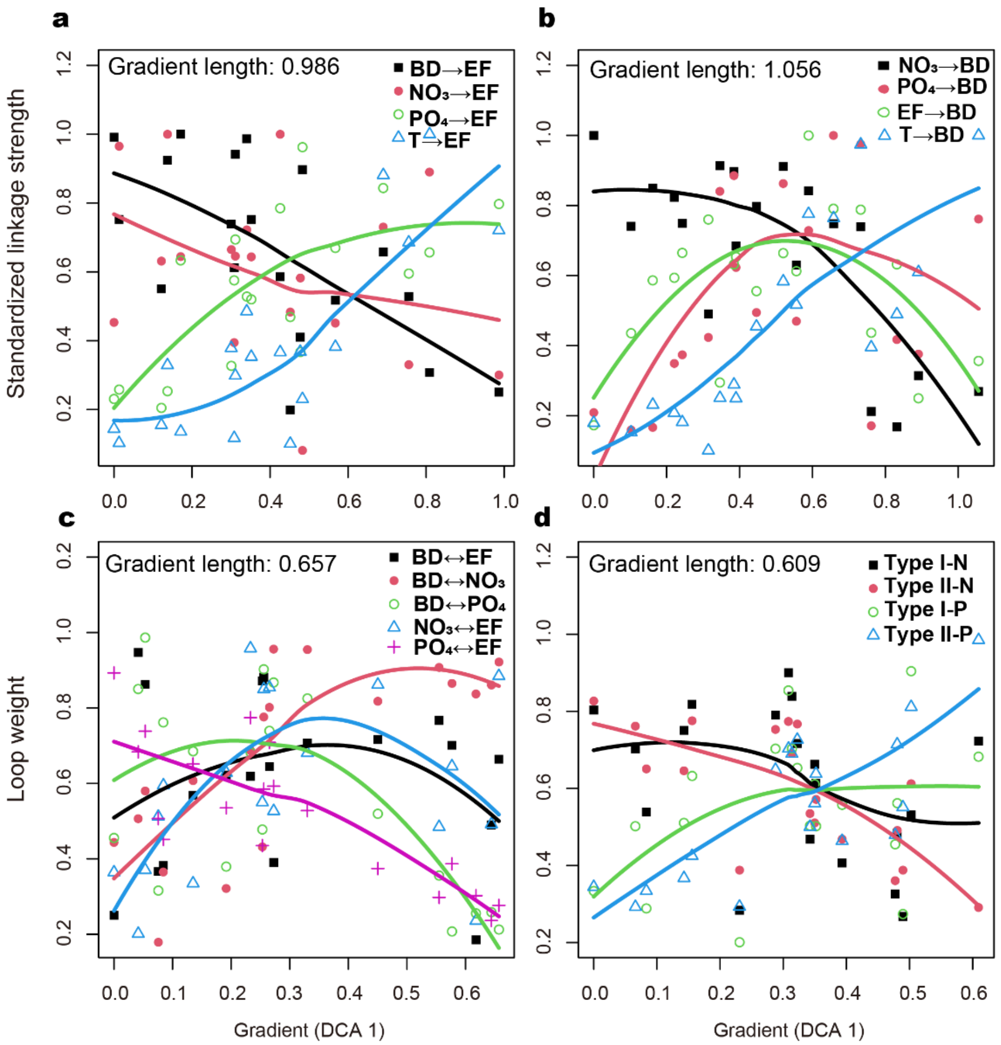

Figure S14 | Justification of direct gradient analysis based on coenoclines.

Coenoclines summarize the quantitative relationships between analysed variables (i.e., linkage strengths or loop weights) and environmental gradients. Specifically, we performed detrended correspondence analysis (DCA) and approximated environmental gradients by the first DCA score, with gradient length defined as the range of scores. For these coenoclines, although there were some unimodal relationships, more linear coenoclines were apparent, due to short gradient lengths. That is, RDA was a more suitable approach than CCA for analysing our dataset, especially when gradient length was short (< 3, as suggested[1]).

Table S1 Basic environmental information for the 16 focal ecosystems. Data are from monthly averaged values. In systems with depth-resolved measurements, data were depth-integrated averages in the euphotic zone; otherwise, only surface-layer measurements were used.

System	Abbrevi-ation	Duration	Area (km^2)	Average depth (m)	Latitude	Average richness	Chl*a* (μg/l)	Temper-ature (˚C)	NO_3 (μg N/l)	PO_4 (μgP/l)	Total phosphorus (μg P/l)
**Lake Biwa**	**Bw**	1978-2010	670	41	35˚20'	17.7	4	14.5	126	1.3	7.5
**Feitsui Reservoir**	**Ft**	1986-2017	10	90	24˚54'	24.2	3.4	24	396	7.7	16.7
**Lake Geneva**	**Gv**	1974-2014	580	153	46˚27'	19.7	4.7	12.1	334	18.9	30.6
**Lake Inba**	**Ib_a; Ib_b; Ib_c**	1986-2016	11.55	1.5	35˚46'	31.3	101.1	16.9	895	11.6	106.5
**Lake Kasumigaura**	**Ks3; Ks9**	1978-2009	220	4	36˚02'	26.1	69.2	16.5	268	11.9	112.1
**Lake Kinneret**	**Kn**	1996-2012	168.7	25.6	32˚49'	38.9	15.4	22.7	90	1.8	19.1
**Lake Maggiore**	**Mg**	1997-2015	212.5	177.5	45˚58'	35.4	3	13.4	759	3.2	7.7
**Lake Mendota**	**Me**	1995-2012	39	13	43˚06'	19.7	8.3	13.6	318	57.5	87.8
**Lake Monona**	**Mo**	1995-2011	13	8	43˚04'	22.4	11	14.2	127	41.7	75.6
**Müggelsee**	**Mu**	1994-2013	7	5	52˚26'	20.2	25.2	11.1	446	65.9	126.4
**Narragansett Bay**	**Ng**	1999-2014	380	9	41˚36'	15.1	7	12	32	20.7	--
**Lake Oneida**	**Od**	1975-1995	207	6.8	43˚10'	26.8	7.5	12.8	218	14.5	36.4
**Shin River**	**Sh**	1986-2016	--	1.7	35˚45'	29.1	96.3	17.4	1989	25.3	117.2
**Lake Võrtsjärv**	**Vo**	2001-2016	270	2.8	58˚18'	27.5	35.6	8.9	568	14	41.9
**Western English Channel**	**Wc**	1992-2009	--	54	50˚15'	27.6	1.5	12.5	48	9.3	--
**Windermere**	**Wd**	1993-2010	15	21.3	54˚21'	25	8.4	11	426	6.1	19

Table S2 Data sources and information for long-term phytoplankton time series.

System	Principal investigator	Contact information	Reference	Counting technique
Lake Biwa	Satoshi Ichise	ichise_home0115@kxf.biglobe.ne.jp	2	Enumerate alive plankton[3]
Feitsui Reservoir	Jiunn-Tzong Wu	jtwu@gate.sinica.edu.tw	4	Modified Utermöhl[4]
Lake Geneva	Orlane Anneville	orlane.anneville@inrae.fr	5	Utermöhl[6]
Lake Inba & Shin River	Maiko Kagami	http://db.cger.nies.go.jp/JaLTER/metacat/metacat/ERDP-2017-04.1/jalter-en	7	Utermöhl[6]
Lake Kasumigaura	Shin-ichiro Matsuzaki	http://db.cger.nies.go.jp/gem/moni-e/inter/GEMS/database/kasumi/	8,9	Utermöhl[6]
Lake Kinneret	Tamar Zohary	tamarz@ocean.org.il	10	Utermöhl[6]
Lake Maggiore	Giuseppe Morabito	https://deims.org/f30007c4-8a6e-4f11-ab87-569db54638fe	11	Utermöhl[6]
Lake Mendota	Stephen Carpenter	https://lter.limnology.wisc.edu/	12	Utermöhl[6]
Lake Monona	Stephen Carpenter	https://lter.limnology.wisc.edu/	13	Utermöhl[6]
Müggelsee	Rita Adrian	adrian@igb-berlin.de	14	Utermöhl[6]
Narragansett Bay	Tatiana Rynearson	http://www.gso.uri.edu/phytoplankton/	15	Sedgewick-Rafter[16]
Lake Oneida	Lars G Rudstam	https://ecommons.cornell.edu/handle/1813/11228	17	Utermöhl[6]
Lake Võrtsjärv	Peeter Nõges	Peeter.Noges@emu.ee	18	Utermöhl[6]
Western English Channel	Claire E. Widdicombe	clst@pml.ac.uk	19	Sedgewick-Rafter[16]
Windermere	Stephen J. Thackeray	sjtr@ceh.ac.uk	20	Utermöhl[6]

Table S3 Availability of time series data among ecosystems.

	Phyto-plankton richness	Chl*a*	Water temperature	NO_3	PO_4	TP	Zoo-plankton	Water column stability	Irradiance
**Lake Biwa**	o	o	o	o	o	o	o	o	o
**Feitsui Reservoir**	o	o	o	o	o	o	-	-	-
**Lake Geneva**	o	o	o	o	o	o	o	o	o
**Lake Inba**	o	o	o	o	o	o	-	-	-
**Lake Kasumigaura**	o	o	o	o	o	o	o	o	o
**Lake Kinneret**	o	o	o	o	o	o	o	o	o
**Lake Maggiore**	o	o	o	o	o	o	o	o	o
**Lake Mendota**	o	o	o	o	o	o	o	-	-
**Lake Monona**	o	o	o	o	o	o	o	-	-
**Müggelsee**	o	o	o	o	o	o	o	o	o
**Narragansett Bay**	o	o	o	o	o	-	-	-	-
**Lake Oneida**	o	o	o	o	o	o	o	o	-
**Shin River**	o	o	o	o	o	o	-	-	-
**Lake Võrtsjärv**	o	o	o	o	o	o	o	o	o
**Western Channel**	o	o	o	o	o	-	-	o	-
**Windermere**	o	o	o	o	o	o	-	o	-

Supplementary References:

1 Šmilauer, P. & Lepš, J. *Multivariate analysis of ecological data using CANOCO 5*. (Cambridge university press, 2014).

2 Hsieh, C. H. *et al.* Phytoplankton community reorganization driven by eutrophication and warming in Lake Biwa. *Aquatic Sciences* **72**, 467-483, doi:10.1007/s00027-010-0149-4 (2010).

3 Kishimoto, N., Ichise, S., Suzuki, K. & Yamamoto, C. Analysis of long-term variation in phytoplankton biovolume in the northern basin of Lake Biwa. *Limnology* **14**, 117-128, doi:10.1007/s10201-012-0390-8 (2013).

4 Wu, J.-T. & Kow, L.-C. Alteration of phytoplankton assemblages caused by changes in water hardness in Feitsui Reservoir, Taiwan. *Bot. Stud.* **51**, 521-529 (2010).

5 Anneville, O. *et al.* Temporal mapping of phytoplankton assemblages in Lake Geneva: annual and interannual changes in their patterns of succession. *Limnology and Oceanography* **47**, 1355-1366 (2002).

6 Utermöhl, H. Zur Vervollkommnung der quantitativen Phytoplankton-Methodik: Mit 1 Tabelle und 15 abbildungen im Text und auf 1 Tafel. *Internationale Vereinigung für theoretische und angewandte Limnologie: Mitteilungen* **9**, 1-38 (1958).

7 Iwayama, A. *et al.* Phytoplankton species abundance in Lake Inba (Japan) from 1986 to 2016. *Ecological Research*, doi:10.1007/s11284-017-1482-z (2017).

8 Takamura, N. & Nakagawa, M. Phytoplankton species abundance in Lake Kasumigaura (Japan) monitored monthly or biweekly since 1978. *Ecological Research* **27**, 837-837, doi:10.1007/s11284-012-0971-3 (2012).

9 Takamura, N., Nakagawa, M. & Hanazato, T. Zooplankton abundance in the pelagic region of Lake Kasumigaura (Japan): monthly data since 1980. *Ecological Research* **32**, 1-1, doi:https://doi.org/10.1007/s11284-016-1406-3 (2017).

10 Zohary, T. Changes to the phytoplankton assemblage of Lake Kinneret after decades of a predictable, repetitive pattern. *Freshwater Biology* **49**, 1355-1371, doi:10.1111/j.1365-2427.2004.01271.x (2004).

11 Morabito, G., Ruggiu, D. & Panzani, P. Recent dynamics (1995-1999) of the phytoplankton assemblages in Lago Maggiore as a basic tool for defining association patterns in the Italian deep lakes. *Journal of Limnology* **61**, 129-145 (2002).

12 Hansen, G. J. A. & Carey, C. C. Fish and phytoplankton exhibit contrasting temporal species abundance patterns in a dynamic north temperate lake. *PLoS ONE* **10**, e0115414, doi:10.1371/journal.pone.0115414 (2015).

13 Vanni, M. J. & Temte, J. Seasonal patterns of grazing and nutrient limitation of phytoplankton in a eutrophic lake. *Limnology and Oceanography* **35**, 697-709, doi:10.4319/lo.1990.35.3.0697 (1990).

14 Wagner, C. & Adrian, R. Consequences of changes in thermal regime for plankton diversity and trait composition in a polymictic lake: a matter of temporal scale. *Freshwater Biology* **56**, 1949-1961, doi:10.1111/j.1365-2427.2011.02623.x (2011).

15 Smayda, T. J. Patterns of variability characterizing marine phytoplankton, with examples from Narragansett Bay. *ICES Journal of Marine Science* **55**, 562-573, doi:10.1006/jmsc.1998.0385 (1998).

16 LeGresley, M. & McDermott, G. Counting chamber methods for quantitative phytoplankton analysis—haemocytometer, Palmer-Maloney cell and Sedgewick-Rafter cell. *Microscopic and molecular methods for quantitative phytoplankton analysis. UNESCO (IOC Manuals and Guides)*, 25-30 (2010).

17 Rudstam, L. G., Mills, E. L., Jackson, J. R. & Stewart, D. J. *Oneida Lake: long-term dynamics of a managed ecosystem and its fishery*. (American Fisheries Society Bethesda, Maryland, USA, 2016).

18 Nõges, P., Mischke, U., Laugaste, R. & Solimini, A. G. Analysis of changes over 44 years in the phytoplankton of Lake Võrtsjärv (Estonia): the effect of nutrients, climate and the investigator on phytoplankton-based water quality indices. *Hydrobiologia* **646**, 33-48, doi:10.1007/s10750-010-0178-y (2010).

19 Widdicombe, C. E., Eloire, D., Harbour, D., Harris, R. P. & Somerfield, P. J. Long-term phytoplankton community dynamics in the western English Channel. *Journal of Plankton Research* **32**, 643-655, doi:10.1093/plankt/fbp127 (2010).

20 Thackeray, S. J., Jones, I. D. & Maberly, S. C. Long-term change in the phenology of spring phytoplankton: species-specific responses to nutrient enrichment and climatic change. *Journal of Ecology* **96**, 523-535, doi:10.1111/j.1365-2745.2008.01355.x (2008).
