# Supplementary material for: Causal networks of phytoplankton diversity and biomass are modulated by environmental context
Source: Nat Commun. 2022 Mar 3;13:1140. doi: 10.1038/s41467-022-28761-3 (PMC8894464; doi:10.1038/s41467-022-28761-3)
Supplement: Supplementary file 3 — Reporting summary [file 41467_2022_28761_MOESM3_ESM.pdf]

nature research

Corresponding author(s): Chih-hao Hsieh

Last updated by author(s): Feb 2, 2022

Reporting Summary

Nature Research wishes to improve the reproducibility of the work that we publish. This form provides structure for consistency and transparency in reporting. For further information on Nature Research policies, see our Editorial Policies and the Editorial Policy Checklist.

Statistics

For all statistical analyses, confirm that the following items are present in the figure legend, table legend, main text, or Methods section.

n/a	Confirmed	
☐	☒	The exact sample size (n) for each experimental group/condition, given as a discrete number and unit of measurement
☐	☒	A statement on whether measurements were taken from distinct samples or whether the same sample was measured repeatedly
☐	☒	The statistical test(s) used AND whether they are one- or two-sided<br>*Only common tests should be described solely by name; describe more complex techniques in the Methods section.*
☐	☒	A description of all covariates tested
☒	☐	A description of any assumptions or corrections, such as tests of normality and adjustment for multiple comparisons
☐	☒	A full description of the statistical parameters including central tendency (e.g. means) or other basic estimates (e.g. regression coefficient) AND variation (e.g. standard deviation) or associated estimates of uncertainty (e.g. confidence intervals)
☐	☒	For null hypothesis testing, the test statistic (e.g. F, t, r) with confidence intervals, effect sizes, degrees of freedom and P value noted<br>*Give P values as exact values whenever suitable.*
☒	☐	For Bayesian analysis, information on the choice of priors and Markov chain Monte Carlo settings
☒	☐	For hierarchical and complex designs, identification of the appropriate level for tests and full reporting of outcomes
☒	☐	Estimates of effect sizes (e.g. Cohen's d, Pearson's r), indicating how they were calculated

Our web collection on statistics for biologists contains articles on many of the points above.

Software and code

Policy information about availability of computer code

Data collection	No software was used to collect the data.
Data analysis	"rEDM" package v1.2.3 (Ye et al. 2020) and "vegan" package v2.5-7 (Oksanen et al. 2020) of R v4.0.3 (R Core Team 2020) was used to analyze the data.

For manuscripts utilizing custom algorithms or software that are central to the research but not yet described in published literature, software must be made available to editors and reviewers. We strongly encourage code deposition in a community repository (e.g. GitHub). See the Nature Research guidelines for submitting code & software for further information.

Data

Policy information about availability of data

All manuscripts must include a data availability statement. This statement should provide the following information, where applicable:

- Accession codes, unique identifiers, or web links for publicly available datasets
- A list of figures that have associated raw data
- A description of any restrictions on data availability

Raw time series datasets from all research sites are available, on request, through the paths listed in Table S2 due to various data use policy. Source data for all figures are provided with this paper and available from Github online repository, https://github.com/biozoo/Chang_etal_2022_SI_CausalFeedback (ref. 83).

Field-specific reporting

Please select the one below that is the best fit for your research. If you are not sure, read the appropriate sections before making your selection.

☐ Life sciences ☐ Behavioural & social sciences ☒ Ecological, evolutionary & environmental sciences

For a reference copy of the document with all sections, see nature.com/documents/nr-reporting-summary-flat.pdf

Ecological, evolutionary & environmental sciences study design

All studies must disclose on these points even when the disclosure is negative.

Study description	The study integrates longterm time series data (16-41 years), including phytoplankton diversity, biomass, and physicochemical factors in 19 monitoring stations, spanning a wide range of freshwater and marine types, from shallow to deep and from oligotrophic to eutrophic. We apply convergent cross mapping (i.e., CCM, a nonlinear time series analysis used to detect causations), to reconstruct quantitative causal networks that consist of key causal links and feedbacks among biodiversity, ecosystem functioning and environmental factors. Through a cross-system comparison, we identify macroecological patterns presenting how causal links and feedbacks vary along environmental gradients.
Research sample	We report two key metrics of phytoplankton communities, including species diversity and biomass. Species richness is used as a proxy of diversity; chlorophyll a concentration is used as a proxy of phytoplankton biomass. In addition, environmental data that critically affect phytoplankton communities are also compiled, including water temperature and nutrients. In some of analyzed systems, the effects of zooplankton, water stability, and light are additionally tested when data are available. All the datasets are from 19 longterm observatory stations monitoring various aquatic ecosystems across nine countries. All these longterm datasets and their associated studies had been individually published. We analyze this comprehensive dataset to quantify the strength of causal interactions among time series variables
Sampling strategy	The sample size of phytoplankton counting leads to the precision in which the range of 95% confidence limit is less than 10% of the average (400~1000 cells per counting). Phytoplankton communities and environmental factors in 19 monitoring stations are regularly sampled for the purposes of water quality monitoring or biodiversity survey over the past decades. We select those time series with time series length > 15 years. For each time series dataset, all the measurements are included to improve the statistical power of CCM analysis in detecting the causations between time series variables. The requirement of time-series length for CCM and S-map was based on previous studies (Sugihara et al. 2012; Chang et al. 2017).
Data collection	The field samples were collected and analyzed by several hundred skilled technicians and researchers over the past decades from different participant countries. However, the samplings follow a very similar procedure often applied to measure key parameters in aquatic ecosystems. For example, standard chemical approaches are applied to measure nutrient and chlorophyll a concentration; Utermöhl method (or relevant approaches) is used to investigate phytoplankton composition in various systems.
Timing and spatial scale	Time series data are integrated with monthly intervals and sampled within the period from 1974 to2016. However, the exact length of sampling period differs among systems (16~41 years). Spatial coverage of the longterm monitoring stations is within 24°N-58°N and 89°W-140°E, including both freshwater and marine environments.
Data exclusions	No data were excluded from our analyses.
Reproducibility	All our main results can be fully reproduced via R code and source data provided at https://github.com/biozoo/Chang_etal_2022_SI_CausalFeedback
Randomization	Randomness existed in CCM analysis when subsampling time series for constructing a library. Other than CCM analysis, randomness was not relevant to our study because we did not randomly allocate samples/organisms/participants into experimental groups.
Blinding	N/A. Blinding was not relevant to our study because we did not allocate samples/organisms/participants into experimental groups.

Did the study involve field work? ☒ Yes ☐ No

Field work, collection and transport

Field conditions	See the details in Supplementary Tables S1
Location	See the details in Supplementary Tables S1
Access & import/export	All the aquatic systems are opened to public, except for Feitsui Reservoir in which the entrance needs the permission from the Taipei Feitsui Reservoir Administration.
Disturbance	Our longterm observatory monitoring did not cause any disturbance.

Reporting for specific materials, systems and methods

We require information from authors about some types of materials, experimental systems and methods used in many studies. Here, indicate whether each material, system or method listed is relevant to your study. If you are not sure if a list item applies to your research, read the appropriate section before selecting a response.

Materials & experimental systems

n/a	Involved in the study
☒	☐ Antibodies
☒	☐ Eukaryotic cell lines
☒	☐ Palaeontology and archaeology
☒	☐ Animals and other organisms
☒	☐ Human research participants
☒	☐ Clinical data
☒	☐ Dual use research of concern

Methods

n/a	Involved in the study
☒	☐ ChIP-seq
☒	☐ Flow cytometry
☒	☐ MRI-based neuroimaging
